# Proteaphagy and the Trafficking of Proteasomes under Nutrient Stress Conditions

**DOI:** 10.1080/27694127.2022.2040764

**Published:** 2022-03-17

**Authors:** Kenrick A Waite, Jeroen Roelofs

**Affiliations:** Department of Biochemistry and Molecular Biology, University of Kansas Medical Center, Kansas City, 3901 rainbow Blvd., HLSIC 1077, Kansas, USA

## Punctum

Proteasomes are found in the cytosol and nucleus of eukaryotic cells, where they are critical for regulating many processes via the timely degradation of — often short-lived — proteins. These multisubunit proteases are abundant and long-lived. Proteasome levels are upregulated under stress conditions when additional proteolytic capacity is required, for example during heat stress. The degradation of proteasome complexes themselves has long received little attention. One of the first indications of the degradation of proteasomes came from a study in 1995 identifying proteasomes in lysosomes of starved rat liver cells, suggesting they were subject to macroautophagy/autophagy. About 20 years later, autophagic degradation of proteasomes regained focus with several groups identifying an evolutionarily conserved, complex, and specifically regulated process of proteasome autophagy, known as proteaphagy. We will discuss our recent paper in this context.

Initially, two forms of proteaphagy were distinguished. The first is triggered when proteasomes are damaged or inhibited with clinically relevant drugs. This process involves the ubiquitination of proteasomes and selective autophagy receptors (Cue5 in yeast, RPN10 in plants) to target proteasomes to phagophores. The second is proteaphagy triggered by nitrogen starvation, a condition that also induces general autophagy. While no selective autophagy receptor has been identified in yeast for starvation-induced proteaphagy, several components dispensable for general autophagy are required for this process. This indicates that proteaphagy is regulated and arguably selective. Consistent with this, upon glucose starvation general autophagy is observed, but no proteaphagy is induced. Proteasomes instead localize to cytosolic condensates known as proteasome storage granules (PSGs). However, in yeast defective for PSG formation, proteasomes become substrates for autophagy. Interestingly, under conditions of low glucose as opposed to no glucose, proteasomes undergo AMPK- and ESCRT-dependent microautophagy. This is a third type of proteaphagy mainly observed for aberrant proteasomes. In all, proteaphagy appears to be fine-tuned to the type of starvation. Therefore, it is important to identify the factors and conditions that influence proteasome trafficking and autophagic degradation.

In our recent publication [1], we tested the prediction that conditions with general autophagy should show robust proteasome autophagy unless PSG formation is triggered. We first focused on rapamycin, as this drug is a strong inducer of general autophagy. While rapamycin has been shown to increase proteasome levels and activity in yeast and mammals, we expected that proteaphagy would be induced because in both nitrogen starvation conditions and upon rapamycin treatment TORC1 inhibition is a major driver of the cellular response. Our data show that proteasome upregulation following rapamycin treatment is transitory and proteaphagy in fact is induced approximately 4 h after the upregulation. This likely reflects a transient need for increased proteasome activity under conditions that inhibit TORC1. This would be consistent with the idea that both proteasomal degradation and autophagy help to resolve amino acid shortages cells may experience. Alternatively, degradation of certain factors by proteasomes early after autophagy induction can facilitate a cell’s adaptive responses. While we do not detect an upregulation of proteasomes following nitrogen starvation, proteaphagy appears slow compared to general autophagy, perhaps reflective of a degradative function early in starvation. Similar responses, albeit somewhat slower, are observed with caffeine, which also inhibits TORC1. Prior to proteaphagy, proteasomes are nuclear, and no proteasome condensates are observed with caffeine, rapamycin, or nitrogen starvation.

**Figure 1.**
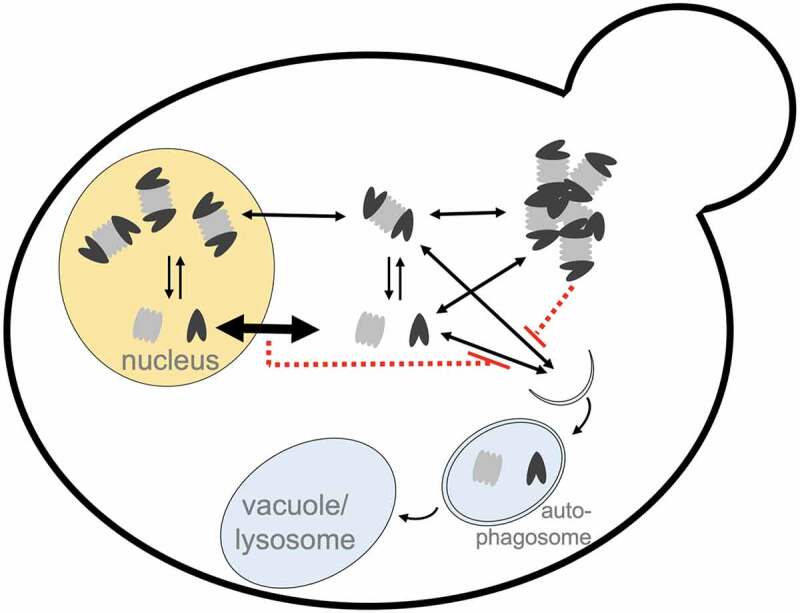
Differential trafficking of proteasomes under nutrient stress. In logarithmically growing yeast most proteasomes reside in the nucleus. Nuclear export can occur for assembled proteasomes but might be more efficient for subcomplexes. Physiological stress, like glucose starvation or prolonged growth to stationary phase, triggers the formation of proteasome condensates (proteasome storage granules, PSGs). Cytosolic and nuclear proteasomes are subject to autophagy during some but not all general autophagy-inducing conditions. Nuclear retention or recruitment to PSGs can protect proteasomes from proteaphagy (indicated by red lines).

Other conditions that induce general autophagy in yeast are amino acid starvation, which induces proteaphagy in mammalian cells, and starvation for phosphate. Under these conditions we detect no and little proteaphagy respectively. Here, the protection from general autophagy is not achieved by PSG formation, as these condensates are not observed. In yeast, most proteasomes are nuclear and nucleaphagy factors such as Nvj1 and Atg39 are not required for proteasome autophagy. Instead, proteasomes require nuclear export to become autophagic substrates. Our amino acid and phosphate starvation data show that keeping proteasomes nuclear also protects them from autophagy. Without a specific autophagy receptor identified, factors that affect proteaphagy can act by two different mechanisms: directly contributing to the process of autophagic packaging of proteasomes or by having an impact on the availability of cytoplasmic proteasomes. The latter can be either through influencing PSG formation or by controlling proteasome nuclear retention or export (Fig. 1). Interestingly, mutations of autophagy genes that prevent proteasome autophagy, for instance in *atg7* deletion strains, result in the nuclear localization of proteasomes in autophagy-inducing conditions. Active autophagy may contribute to the process of proteasome nuclear export.

How proteasome nuclear export is regulated remains poorly understood and it might occur at different efficiencies for assembled proteasomes versus subcomplexes. Consistent with this, it has been shown that as a pre-requisite to their autophagic degradation, nuclear proteasome complexes dissociate into subassemblies: core and regulatory particles. Both subassemblies require different factors for efficient proteaphagy. For example, we observed that the selective autophagy receptor Atg11 is required for the autophagic degradation of a subset of proteasome regulatory particles upon nitrogen starvation but is not involved in proteaphagy of core particles. Proteasome dissociation allows for additional regulation of nuclear export by controlling the level of proteasome holo-complexes versus subassemblies, as well as at the export of these subassemblies (Fig. 1). Interestingly, we identified the MAP kinase Slt2/Mpk1 (and kinases directly upstream) as signaling molecules required for efficient proteaphagy. In kinase mutant strains we observe proteasome nuclear retention. As deletion of *SLT2* does not inhibit general autophagy, we hypothesize this kinase regulates a step required for proteasome nuclear export.

The recent studies of proteasome trafficking via autophagy or to cytosolic condensates have revealed a surprisingly complex and nuanced process. The nuclear retention of proteasomes in some autophagy-inducing conditions might indicate a need for nuclear proteasome activity. Conversely, proteasome sequestration into PSGs might reflect a critical need for reduced nuclear proteasome activity. In all, work from us and many other labs (much of which we were unable to highlight in this commentary) outline a complex regulatory network acting on proteasomes when cells are stressed. We expect this reflects important physiological functionality that contributes to cellular fitness, which the field is beginning to unravel.

## References

[1] Waite AK, Burris A, Vontz G, Lang A, Roelofs J. Proteaphagy is specifically regulated and requires factors dispensable for general autophagy J Biol Chem. 2022 Jan;298(1):101494. doi: 10.1016/j.jbc.2021.101494. PMID: 34919962 34919962 PMC8732087

